# *HLA-DRB1* Shared Epitope-Dependent DR-DQ Haplotypes Are Associated with Both Anti-CCP–Positive and –Negative Rheumatoid Arthritis in Chinese Han

**DOI:** 10.1371/journal.pone.0071373

**Published:** 2013-08-12

**Authors:** Xu Liu, Jianping Guo, Yuan Jia, Yi Zhao, Xia Liu, Feng Cheng, Xiaoxia Li, Yi Zheng, Xuhua Shi, Haiyun Li, Cibo Huang, Yongjing Cheng, Bei Lai, Yanhong Huang, Tian Wang, Bo Ding, ZhangGuo Li

**Affiliations:** 1 Department of Rheumatology and Immunology, Peking University People's Hospital, Beijing, China; 2 Department of Rheumatology, Xuanwu Hospital Capital Medical University, Beijing, China; 3 Department of Rheumatology, China-Japan Friendship Hospital, Chaoyang District, Beijing, China; 4 Institute of Vegetables and Flowers, Chinese Academy of Agricultural Sciences, Haidian District, Beijing, China; 5 Department of Rheumatology, Chao-yang Hospital, Chaoyang District, Beijing, China; 6 Department of Rheumatology, Beijing Hospital of the Ministry of Health, Beijing, China; 7 Department of Rheumatology, Beijing Jishuitan Hospital, Beijing, China; 8 Department of Internal Medicine, Beijing Anzhen Hospital Capital Medical University, Beijing, China; 9 Institute of Environmental Medicine, Karolinska Institute, Stockholm, Sweden; University of Southern California, United States of America

## Abstract

The association between Human Leukocyte Antigen (HLA) class II and rheumatoid arthritis (RA) has been extensively studied, but few reported DR-DQ haplotype. Here we investigated the association of *HLA-DRB1*, *DQA1*, *DQB1*, and DR-DQ haplotypes with RA susceptibility and with anti-CCP antibodies in 281 RA patients and 297 control in Han population. High-resolution genotyping were performed. The *HLA-DRB1* shared epitope (SE)-encoding allele *0405 displayed the most significant RA association (*P* = 1.35×10^−6^)**.** The grouped *DRB1* SE alleles showed great association with RA (*P* = 3.88×10^−13^). The *DRB1* DRRAA alleles displayed significant protective effects (*P* = 0.021). The SE-dependent DR-DQ haplotype SE-DQ3/4/5 remained strong association with both anti-CCP -positive (*P* = 3.71×10^−13^) and -negative RA (*P* = 3.89×10^−5^). Our study revealed that SE alleles and its haplotypes SE-DQ3/4/5 were highly associated with RA susceptibility in Han population. The SE-DQ3/4/5 haplotypes were associated with both anti-CCP positive RA and -negative RA.

## Introduction

Rheumatoid arthritis (RA) is characterized by chronic inflammation of synovial joints, resulting in progressive destruction of cartilage and bone. Both genetic and environmental factors contribute to development of RA. The most important genetic factors associated with RA are the human leukocyte antigen (HLA) linked genes, accounting for approximately 30% of the total genetic contribution for RA susceptibility [1], [2]. Of which, the increase in frequencies of HLA-DRB1*0101, *0102, *0401, *0404, *0405, *0408, *1001 and *1402 were reported in RA patients in different ethnic groups. These *HLA-DRB1* alleles encode a conserved amino acid sequence (^70^Q(R)K(R)RAA^74^), termed the shared epitope (SE) and seemed to be the most recognized and powerful RA genetic risk factors [3]. The DRB1*0901 allele, a corresponding motif ^70^RRRAE^74^, was rare in White and African populations but very frequent among Asians[4]–[6]. Besides the *HLA–DRB1* alleles that contribute to RA susceptibility, certain *HLA–DRB1* alleles conferred protective effects against RA. The best known *DRB1* protective alleles harbored a unique amino acid sequence at position 70 (D^70^ alleles) [7], among which both DRRAA and DERAA conferred significant protective effects.

In addition to *DRB1*, the HLA-DQ molecules may also play a role in RA susceptibility. Experimental studies in transgenic mice have suggested that HLA-DQ was predisposes to arthritis and could modulate disease severity by being source of self-peptides presented in the context of DR [8], [9] . In human, it has been reported that DQA1*03-DQB1*03 (DQ3) homozygous predisposed more strongly to RA and to a more severe disease while DQA1*01-DQB1*0501 (DQ5) homozygous was weakly associated with RA and often with a mild form of undifferentiated arthritis [10] . The haplotype DQ3 linked to DRB1*0901 or *04 and the haplotype DQ5 linked to DRB1*0101, *0102, *0103, and *1001 were positively associated with RA in Caucasians [11], [12] . However, the contribution of DQ genes to RA is undistinguishable from *DRB1*, due to the strong linkage disequilibrium (LD).

Even though the well known HLA-DRB1 has the strongest genetic effect in RA, the DR-DQ haplotypes, as well as its relation with RA patients were unknown. It has been shown that the *HLA-DRB1* genetic background was more specific for association with anti-cyclic citrullinated peptides (anti-CCP) positive RA [13]. However, the interaction of HLA-DR-DQ haplotype with anti-CCP in RA susceptibility remains to be undetermined so far. Furthermore, few RA association studies have been so far performed in Chinese Han population. In present study, we aimed to clarify the contribution of *HLA-DRB1, DQA1*and *DQB1* alleles and DR-DQ haplotypes to RA susceptibility in Han population, and to further determine whether certain DR-DQ haplotypes were specifically associated with RA subsets, e.g. anti-CCP positive/nagetive RA.

## Materials and Methods

### Study Subjects

A total of 281 RA patients were recruited from the Department of Rheumatology Peking University People’s Hospital (mean onset age 40.2±13.6 years, 76.4% females). All patients satisfied the American College of Rheumatology 1987 revised criteria for a diagnosis of RA [14] . The data regarding anti-CCP antibodies were available in 204 RA patients (75.0% anti-CCP-positive, n = 153; 25.0% anti-CCP-negative, n = 51), and the data regarding rheumatoid factor (RF) were available in 128 RA patients (71.1% RF-positive, n = 91; 28.9% RF-negative, n = 37). The control group comprised 297 non-related healthy individual (mean age 42.6±8.6 years; 77.7% females) and was recruited from Health Care Center from Peking University People Hospital.

All patients and healthy controls were self-reported Han Chinese originated from the region of northern China. The study was approved by the medical ethics committee of Peking University People’s hospital and the written informed consents were obtained from all participants to publish these case details.

### HLA Genotyping

The *HLA-DRB1*, *DQA1* and *DQB1* were genotyped by using sequence based typing (SBT). The strategy of both DR and DQ included forward and backward amplification and sequencing of exons 2. The amplification primers for exon 2 were designed on the basis of known intron sequences [15]. Alleles which cannot be separated by exon2 were group together, e.g. HLA-DQA1*03. The sequences of *HLA-DRB1, DQA1* and *DQB1* were analyzed using Assign software (UTYPE, Invitrogen), which enables assignment of genotypes based on a recent library file of HLA alleles. Ambiguous alleles of *HLA-DRB1* were additionally performed by sequence-specific polymerase chain reaction, according to the reference protocol (Invitrogen 45040-4). The time resolved fluorescence hybridization was performed for the ambiguous alleles of *DQA1* and *DQB1* [16]. All samples were genotyped for *DRB1*. The *DQA1* and *DQB1* typing was performed in 269 RA patients and 297 controls.

### Anti-CCP Antibody Detection

Anti-CCP antibodies were quantitatively tested using the second generation kit by an enzyme-linked immunosorbent assay (ELISA; Euroimmun, Germany). A cutoff value of 5 relative units (RU/ml) was established as recommended by the manufacturer’s protocol.

### Haplotype Computational Estimation

Molecular haplotyping required family-based data to establish phases. For our phase-unknown population-based data, the haplotypes were statistically calculated by using software Arlequin 3.1 (http://cmpg.unibe.ch/software/arlequin3/). The Hardy-Weinberg Equilibrium (HWE) was calculated locus by locus and for whole haplotype. All variants were in HWE in whole cohort (*P*>0.05, data not shown). The Markov chain approximation was used with 100000 steps and 1000 dememorization steps definition. The distribution of HLA-DQ-DR haplotypes was analyzed in all patients and controls, using pseudo-Bayesian based ELB approach [17]. The software Arlequin 3.1 was run by using the following settings: ε = 1e−7; 5 significant digits for output 50 starting points for ELB algorithm and a maximum of 1000 iterations.

### Statistical Analysis

The differences of allelic/haplotypic distribution between cases and controls were analyzed using chi-square test or Fisher’s exact test (when frequency <5), with two-tailed *P* values. Odds ratios (ORs) were calculated with 95% confidence intervals (95% CIs) in 2×2 tables. The chi-square values for the individual alleles were determined after stratifying the data using the relative predispositional effect (RPE) method [18]. Statistical analyses were performed with SPSS version 13.0 software. *P* values <0.05 were considered statistically significant.

## Results

### Association of HLA-DRB1, -DQA1 and –DQB1 with RA in Han Population

To clarify the haplotype association of HLA-DR-DQ with RA, first the frequencies of each HLA-DRB1, -DQA1 and -DQB1 allele were measured by sequence based typing for 281 RA patients and 297 ethnically matched healthy controls. A total of 45 HLA–DRB1, 10–DQA1 and 13–DQB1 alleles were identified. The alleles with frequencies more than 1% in cases or controls were listed in Table 1 and Table 2.

**Table 1:** Association of *HLA-DRB1* alleles with RA by RPE method in 281 patients and 297 controls.

HLA-DRB1	70–74	RA	Control	OR (95%CI)	*P* value*
	motif	n (%)	n (%)		
**SE alleles**		134(23.8)	49 (8.2)	3.48 (2.45–4.95)	3.88×10^−13^
0101	QRRAA	25 (4.4)	8 (1.3)	3.77 (1.68–8.43)	1.00×10^−3^
0404	QRRAA	8 (1.4)	2 (0.3)	−	0.049^§^
0405	QRRAA	53 (9.4)	16 (2.7)	3.76 (2.12–6.66)	1.35×10^−6^
0410	QRRAA	11 (1.8)	0 (0.0)	−	3.82×10^−4§^
0401	QKRAA	18 (3.2)	11 (1.9)	2.01 (0.94–4.30)	0.067
1001	RRRAA	19 (3.4)	12 (2.0)	1.95 (0.94–4.05)	0.070
**DRRAA alleles**		92(16.4)	129(21.7)	0.71 (0.52–0.95)	0.021
1101	DRRAA	24 (4.3)	35 (5.9)	−	−
1201	DRRAA	28 (5.0)	30 (5.1)	−	−
1202	DRRAA	23 (4.1)	48 (8.1)	0.49 (0.29–0.81)	5.00×10^−3^
1602	DRRAA	17 (3.0)	16 (2.7)	−	−
**Others**					
1302	DERAA	5 (0.9)	12 (2.0)	0.40 (0.14–1.14)	0.076
0701	DRRGQ	49 (8.7)	70 (11.8)	−	−
0803	DRRAL	17 (3.0)	20 (3.4)	−	−
0301	QKRGR	20 (3.6)	28 (4.7)	−	−
0403	QRRAE	7 (1.2)	6 (1.0)	−	−
0901	RRRAE	94 (16.7)	86 (14.5)	−	−
1401	RRRAE	10 (1.8)	16 (2.7)	−	−
1405	RRRAE	8 (1.4)	15 (2.5)	−	−
1501	QARAA	81 (14.4)	96 (16.2)	−	−
1502	QARAA	12 (2.1)	12 (2.0)	−	−

Alleles with frequencies less than 1% in both cases and controls were not listed.

RPE: relative predispositional effect; *P<0.05 were listed; ^§^Fisher's exact test.

RA: rheumatoid arthritis; OR (95% CI): odds ratio (95% confidence interval).

SE: Shared Epitope QR(K)RAA or RRRA.

**Table 2:** Association of *HLA-DQ* alleles with RA by RPE method.

HLA-DQ	RA	Control	OR (95%CI)	*P* value*
	n (%)	n (%)		
***DQA1***				
03*	189 (35.1)	152 (25.6)	1.56 (1.22–2.03)	4.76×10^−4^
0102	85 (15.8)	115 (19.4)	−	−
0501/03/05#	74 (13.8)	107 (18.0)	−	−
0101/04/05#	71 (13.2)	69 (11.6)	−	−
0201	47 (8.7)	61 (10.3)	−	−
0103	41 (7.6)	38 (6.4)	−	−
0601	27 (5.0)	51 (8.6)	0.56 (0.35–0.91)	0.018
***DQB1***				
0303	104 (19.3)	91 (15.3)	1.44 (1.05–1.96)	0.022
0501	83 (15.4)	65 (10.9)	−	−
0502	6 (1.1)	11 (1.9)	−	−
0503	5 (0.9)	13 (2.2)	−	−
0301	99 (18.4)	150 (25.3)	0.67 (0.50–0.89)	5.00×10^−3^
0601	93 (17.3)	126 (21.2)	0.68 (0.50–0.92)	0.013
0201	65 (12.1)	78 (13.1)	−	−
0401	51 (9.5)	21 (3.5)	2.86 (1.70–4.82)	4.27×10^−5^
0302	30 (5.6)	41 (6.9)	−	−

Alleles with frequencies less than 1% in both cases and controls were not listed.

RPE: relative predispositional effect; * *P*<0.05 were listed.

# alleles that could not be identified by SBT of exon 2.

RA: rheumatoid arthritis; OR (95% CI): odds ratio (95% confidence interval).

Significant RA association were observed with –DRB1 alleles *0101, *0404, *0405 and *0410, compared with healthy controls (Table 1, *P* = 1.00×10^−3^, 0.049, 1.35×10^−6^ and 3.82×10^−4^, respectively), which was in concordance with the results from other Asian populations [4]. All the susceptible alleles had QRRAA motif at 70–74 amino acid position resided in HLA-DRB1. Unlike in Caucasians [3], DRB1*0401 encoding QKRAA at 70–74 amino acid was not associated with RA in our study cohort (*P* = 0.067). When the SE alleles were grouped, as shown in Table1, it showed a strong association with RA susceptibility in Han population (*P* = 3.88×10^−13^). In contrast, the allele DRB1*1202 and *1302 which encode DRRAA and DERAA had higher frequencies in healthy controls than that in RA patients (*P* = 5.00×10^−3^ and 0.076, respectively). When the DRRAA alleles were grouped, its RA protective effects still remained (*P* = 0.021, Table 1).

HLA-DQ typing was performed in 267 RA patients and 297 controls. As shown in Table 2, the alleles DQA1*03, DQB1*0303 and DQB1*0401 were significantly associated with RA susceptibility (*P* = 4.76×10^−4^, 0.022 and 4.27×10^−5^, respectively). Alleles DQA1 *0601, DQB1*0301 and *0601 displayed protective effects against RA (*P* = 0.018, 0.005 and 0.013, respectively). The DQA1-DQB1 haplotype was also calculated. As shown in Table 3, the haplotype DQA1*03-DQB1*0303(DQ3) and DQA1*03-DQB1*0401(DQ4) displayed increased susceptibility to RA (*P* = 0.042 and 5.93×10^−5^, respectively), whereas the haplotype DQA1*0601-DQB1*0301 (DQ3) and DQA1*0102-DQB1*0601(DQ6) displayed protective effects (*P* = 0.010 and 0.049, respectively). However, as the same serotype were grouped, unlike in Caucasians neither DQ3 nor DQ5 alleles were associated with RA (*P* = 0.15 and 0.13, respectively).

**Table 3:** Association of DQA1-DQB1 haplotype with RA susceptibility.

DQA1-DQB1	Serotype^†^	RA	Control	OR (95%CI)	*P* value*
		n (%)	n (%)		
**Total _DQ2_**		61 (11.3)	73 (12.3)	−	−
0201-0201	DQ2	37 (6.9)	−	−	−
03-0201	DQ2	7 (1.3)	−	−	−
0501-0201	DQ2	17 (3.2)	−	−	−
**Total _DQ3_**		220 (40.9)	268 (45.1)	−	−
03-0301	DQ3	20 (3.7)	−	−	−
0501-0301	DQ3	52 (9.7)	−	−	−
0601-0301	DQ3	24 (4.5)	49 (8.2)	0.52 (0.31–0.86)	0.010
03-0302	DQ3	24 (4.5)	39 (6.6)	−	−
0102-0303	DQ3	6 (1.1)	6 (1.0)	−	−
0201-0303	DQ3	7 (1.3)	11 (1.9)	−	−
03-0303	DQ3	87 (6.2)	71 (12.0)	1.42 (1.01–2.00)	0.042
**Total _DQ4_**					
03-0401	DQ4	46 (8.6)	18 (3.0)	2.99 (1.71–5.23)	5.93×10^−5^
**Total _DQ5_**		76 (14.1)	66 (11.1)	−	−
0101-0501	DQ5	61 (11.3)	50 (8.4)	−	−
0102-0501	DQ5	15 (2.8)	16 (2.7)	−	−
**Total _DQ6_**		74 (13.8)	98 (16.5)	−	−
0103-0601	DQ6	35 (6.5)	35 (5.9)	−	−
0102-0601	DQ6	39 (7.2)	63 (10.6)	0.66 (0.43–1.00)	0.049

Alleles with frequencies less than 1% in both cases and controls were not listed.

* *P*<0.05 were listed.

RA: rheumatoid arthritis; OR (95% CI): odds ratio (95% confidence interval).

### The SE-grouped but not DQ3/DQ5-grouped HLA-DR-DQ Haplotypes Remained Predominant Association with RA

HLA DR and DQ are in strong linkage disequilibrium [19] , therefore the association of DR-DQ haplotypes with RA susceptibility was calculated. The frequencies of the DR-DQ haplotypes were shown in table 4. All susceptibility haplotypes were SE-related. The highest RA risk was associated with the haplotypes QRRAA-DQ4 (DRB1*0405/10- DQA1*03–DQB1*0401, *P* = 3.05×10^−6^).

**Table 4:** Association of HLA-DR-DQ halotypes with RA stratified by *DRB1* 70–74 motif.

DRB1	DQ	DR-DQ Haplotype	RA	Control	OR (95%CI)	*P* value*
70–74 motif			n (%)	n (%)		
**Grouped by SE**			101 (18.8)	32 (5.4)	4.06 (2.68–6.16)	2.85×10^−12^
QKRAA	DQ3	0401-(03)-0301/02/03	14 (2.6)	10 (1.7)	−	−
QRRAA	DQ3	0404/05/10-(03)-0301/02/03	20 (3.7)	5 (0.8)	4.55 (1.70–12.2)	0.010
QRRAA	DQ4	0405/10-(03)-0401	43 (8.0)	12 (2.0)	4.21 (2.20–8.08)	3.05×10^−6^
QRRAA	DQ5	0101/02-0101-0501	24 (4.5)	5 (0.8)	5.50 (2.08–14.50)	1.19×10^−4^
RRRAA	DQ5	1001-0101-0501	18 (3.3)	8 (1.3)	−	−
**Grouped by DRRAA**			72 (13.4)	123 (20.7)	0.59 (0.43–0.81)	1.00×10^−3^
DRRAA	DQ3	1101-0501-0301	21 (3.9)	35 (5.9)	−	−
DRRAA	DQ3	1101-0102-0303	0 (0.0)	6 (1.0)	−	−
DRRAA	DQ3	1101-0501-0303	1 (0.2)	11 (1.9)	0.10 (0.01–0.77)	6.00×10^−3^
DRRAA	DQ3	1201-0501-0301	21 (3.9)	17 (2.9)	−	−
DRRAA	DQ3	1202-0601-0301	19 (3.5)	45 (7.6)	0.45 (0.26–0.77)	0.030
DRRAA	DQ5	1602-0102-0501	10 (1.9)	9 (1.5)	−	−
**Grouped by RRRAE**			95 (17.7)	92 (15.5)	−	−
RRRAE	DQ3	0901-(03)-0302/03	83 (15.4)	78 (13.1)	−	−
RRRAE	DQ5	1405-0101-0501	6 (1.1)	7 (1.2)	−	−
RRRAE	DQ5	1401-0101-0501	6 (1.1)	7 (1.2)	−	−
**Others**						
DERAA	DQ6	1302-0102-0601	4 (0.7)	6 (1.0)	−	−
DRRGQ	DQ3	0701-0201-0303	7 (1.3)	11 (1.9)	−	−
DRRGQ	DQ2	0701-0201-0201	35 (6.5)	44 (7.4)	−	−
DRRAL	DQ6	0803-0103-0601	15 (2.8)	18 (3.0)	−	−
QRRAE	DQ3	0403-(03)-0302	5 (0.9)	4 (0.7)	−	−
QRRAE	DQ3	0406-(03)-0302	2 (0.4)	11 (1.9)	−	−
QARAA	DQ3	1501-0102-0303	5 (0.9)	3 (0.5)	−	−
QARAA	DQ6	1501-0102-0601	44 (8.2)	69 (11.6)	−	−
QARAA	DQ6	1501-0103-0601	14 (2.6)	4 (0.7)	−	−
QARAA	DQ6	1502-0103-0601	4 (0.7)	7 (1.2)	−	−
QKRGR	DQ2	0301-0501-0201	18 (3.3)	24 (4.0)	−	−

Alleles with frequencies less than 1% in both cases and controls were not listed.

* *P*<0.05 were listed.

RA: rheumatoid arthritis; OR (95% CI): odds ratio (95% confidence interval);

SE: Shared Epitope QR(K)RAA or RRRAA.

To test whether there is an independent effect of HLA-DRB1 on RA susceptibility, we stratified the DR-DQ haplotypes by DRB1 and DQ separately. When the haplotypes were stratified by SE status, it remained strong association with RA susceptibility independent of DQ status (Table 4, *P* = 2.85×10^−12^). Unlike SE alleles, when the haplotypes were grouped according to DQ status, there were no association observed between DQ2/D3/D5/D6-haplotypes and RA susceptibility except for the DQ4- related ones (Table 5), indicating DRB1 may provides more contribution in RA genetics than DQ does. Further, when the haplotypes were grouped according to DRRAA status, a strong RA protective effect was observed (*P* = 0.001, Table 4).

**Table 5:** Association of HLA-DR-DQ halotypes with RA stratified by DQ.

Haplotypes	RA	Control	OR(95%CI)	*P* value*
	n (%)	n (%)		
DR-DQ2†	61 (11.3)	73 (12.3)	−	−
DR-DQ3	220 (40.9)	268 (45.1)	−	−
DR-DQ4	46 (8.6)	18 (3.0)	2.99 (1.71–5.23)	5.93×10^−5^
DR-DQ5	76 (14.1)	66 (11.1)	−	−
DR-DQ6	74 (13.8)	98 (16.5)	−	−

* *P*<0.05 were listed.

RA: rheumatoid arthritis; OR (95% CI): odds ratio (95% confidence interval).

† DQ2:DQA1*0201/03/0501-DQB1*0201; DQ3: DQA1*03-DQB1*0301/02/03; DQ4: DQA1*03 -DQB1* 0401; DQ5: DQA1*0101/04-DQB1*0501; DQ6: DQA1*0102/03-DQB1*0601;

### SE-related DR–DQ Haplotypes were Associated with both Anti-CCP Positive and Anti-CCP Negative RA, whereas DRRAA-related DR–DQ Haplotypes were Protective against Anti-CCP Positive RA

Due to the large number of DR–DQ haplotypes and the independence of the 70–74 motif of *DRB1* contributed to RA susceptibility, we focused on specific DR–DQ haplotypes to reduce the complexity of analysis. The patients having anti-CCP antibodies data available were grouped based on the presence of SE-, DRRAA and RRRAE-encoding alleles. As shown in Table 6, following stratification for anti-CCP status, we found the SE–DQ3/DQ4/DQ5 haplotypes showed strong association not only with anti-CCP positive RA (*P* = 3.71×10^−13^), but also with anti-CCP negative RA despite reduced power in the analysis (*P* = 3.89×10^−5^). Conversely, the DRRAA- DQ3/DQ5 haplotypes displayed strong protective effects against anti-CCP positive RA (*P* = 0.010) but not for anti-CCP negative RA (*P* = 0.800, data not shown). Interestingly, in general, the RRRAE-DQ3 haplotypes was not associated with susceptibility to RA. However, following anti-CCP stratification, we found the RRRAE-DQ3 haplotypes conferred an increased susceptibility to anti-CCP positive RA (*P* = 0.029). A similar association pattern was observed in terms of RF. SE–DQ3/DQ4/DQ5 haplotypes showed strong association with both RF-positive and -negative RA (*P* = 4.47×10^−12^ and 8.08×10^−5^, respectively). The RRRAE-DQ3 haplotypes were also showed association with RF-positive RA, though did not reach statistical significance (*P* = 0.062). Accordingly, the DRRAA- DQ3/DQ5 haplotypes were displayed protective effects against RF-positive RA (*P* = 0.078), though the difference did not reach statistical significance.

**Table 6:** Association of HLA-DR-DQ halotypes with RA stratified by anti-CCP antibodies and RF status.

	Haplotype	n (%)	OR (95%CI)	*P* value*
#SE-DQ3/DQ4/DQ5				
	Anti-CCP positive	65 (21.3)	4.74 (3.02–7.42)	3.71×10^−13^
	Anti-CCP negative	17 (16.7)	3.51 (1.87–6.60)	3.89×10^−5^
	RF positive	42 (22.3)	5.05 (3.08–8.29)	4.47×10^−12^
	RF negative	13 (17.6)	3.74 (1.86–7.51)	8.08×10^−5^
	Control	32 (5.4)	−	−
§RRRAE-DQ3				
	Anti-CCP positive	57 (18.6)	1.51 (1.04–2.20)	0.029
	Anti-CCP negative	17 (16.7)	−	−
	RF positive	35 (18.6)	1.51 (0.98–2.34)	0.062
	RF negative	11 (18.6)	−	−
	Control n (%)	78 (13.1)	−	−
‡DRRAA-DQ3/DQ5				
	Anti-CCP positive	42 (13.7)	0.61 (0.42–0.89)	0.010
	Anti-CCP negative	18 (17.6)	−	−
	RF positive	28 (14.9)	0.67 (0.43–1.05)	0.078
	RF negative	15 (20.3)	−	−
	Control n (%)	123 (20.7)	−	−

* *P*<0.05 were listed.

RA: rheumatoid arthritis; anti-CCP antibodies: anti-cyclic citrullinated peptides antibodies; RF: rheumatoid factor.

OR (95% CI): odds ratio (95% confidence interval).

# 0401/04/05/10-(03)-0301/02/03/0401; and 0101/02/1001-0101-0501.

§ 0901-(03)- 0302/03.

‡ 1101/1104/1201/1202/1601/1602-0102/0501/0601-0301/02/03.

## Discussion

In this study, we found that the most significant DRB1 allele in susceptibility to RA in Han population was DRB1*0405 encoding QRRAA, a finding that is consistent with previous studies in other Asian populations [4], [5]. When the SE alleles were grouped, it showed a strong association with RA susceptibility. The DRRAA alleles displayed protective effects against RA in Han population. Among the three HLA loci, DRB1 provided more contribution in RA susceptibility than DQ did. Furthermore, for the first time, we showed that the presence of SE-DQ3/4/5 haplotype was strongly associated with both anti-CCP positive and anti-CCP negative RA.

DRB1*0901, encoding RRRAE at position 70–74, has been reported as susceptible allele for RA in several Asian populations[4]–[6]. In the present study, at allele level we didn’t find significant association between DRB1*0901 and RA, despite the allelic frequency of *0901 in our control group was similar with other Asian ethnic groups [5], [6] **.** However, at haplotype level, *0901-DQ3 conferred susceptibility to anti-CCP positive RA. The result indicated that the interaction between DRB1*0901 and DQ3 may contribute to RA susceptibility in Han population. In Caucasians, the haplotypes grouped by QKRAA-DQ3 were RA susceptible, with high haplotypic frequencies [12]. However, QKRAA-DQ3 displayed no association with RA in our cohort, with much lower haplotypic frequencies (Table 4).

Previously Lee, *et al.* has reported that DRB1*1302 was the strongest RA protective allele in Korean population [5]. However, we found that DRB1*1302 encoding DERAA which was crucial in RA protection (RAP) model [20], was rather rare in our study cohort (0.9% in RA and 2.0% in control, *P* = 0.076). The protective allele DRB1*1202 encoding DRRAA was more frequent than DRB1*1302 and conferred a significant RA protective effect. The grouped *DRB1* DRRAA-DQ3/DQ5 haplotypes displayed protective effects against anti-CCP positive RA.

Several studies have reported that SE-encoding HLA-DRB1 alleles were only associated with anti-CCP positive RA but not with anti CCP negative RA in Caucasians and Asians[21]–[23]. Furthermore, there has been so far no any study describing relationship between ACPA and DR-DQ haplotype. In this work, we showed that SE-DQ3/DQ4/DQ5 haplotypes were associated not only with anti-CCP positive RA also with anti CCP negative RA. In terms of RF, a similar association pattern was observed. Recently, Mackie SL *et al*. also found that association of SE with both anti-CCP positive and negative RA in a large UK population [24]. Future studies including greater numbers of study subjects are needed to further clarify this effect.

In conclusion, we demonstrated that SE alleles and its haplotypes SE-DQ3/DQ4/DQ5 were highly associated with RA susceptibility in Han population. The DRB1- DRRAA alleles and its haplotypes DRRAA-DQ3/DQ5 displayed protective effects against RA. The HLA DR-DQ haplotypes containing RA susceptible or protective alleles were mainly associated with anti-CCP positive RA. However, the SE-DQ3/DQ4/DQ5 haplotypes were also associated with anti-CCP negative RA.
